# Pregnancy-associated-cancer in the French West Indies (Martinique): maternal and neonatal outcomes

**DOI:** 10.1186/s12884-017-1524-0

**Published:** 2017-10-02

**Authors:** Kathleen Melan, Jean-Luc Volumenie, Gaël Wan-Ajouhu, Stephen Ulric-Gervaise, Jacqueline Veronique-Baudin, Clarisse Joachim

**Affiliations:** 1 AMREC, Fort-de-France, Martinique France; 2 Gynaecology, Obstetrics Department, University Hospital of Martinique, Fort-de-France, Martinique France; 3 Oncology Haematology Urology Pathology Department, University Hospital of Martinique, UF 1441 Cancer Research and Registry, 127 Route de Redoute, Les jardins de la Mouïna, 97200 Fort-de-France, Martinique France

**Keywords:** Cancer in pregnancy, Management, Treatment, Pregnancy outcomes

## Abstract

**Background:**

The management of pregnancy-associated-cancer (PAC) requires epidemiological evaluation of the pathways of care. The aim of this study was to describe maternal and neonatal outcomes of PAC in Martinique.

**Methods:**

A retrospective study was conducted using data from medical records and the Martinique Cancer Registry for all PAC diagnosed between 1st January 2000 and 31st December 2014.

**Results:**

Eighteen women were diagnosed with PAC: 17 during pregnancy and one during the postpartum period. Mean age at diagnosis was 35.7 ± 5.4 years. PAC were mainly gynecological cancers (12/18); the other sites were: lymphoma, brain, liver, colon, skin and unknown primary site. In most cases, PAC was detected in symptomatic individuals (72.2%). Nine women had nodal involvement or initial metastasis at diagnosis. No chemotherapy was administered in cases of preservation of pregnancy. Seven fetal losses caused by abortion and miscarriage were recorded, and 11 women conducted viable pregnancies. The main neonatal pathology observed was prematurity (58.3%).

**Conclusion:**

Cancer management during pregnancy is a challenge for French West-Indies territories. A Caribbean Observatory of rare cancers could help to ensure a coordinated approach to support and monitoring for these patients.

## Background

In 2013, more than 12,800 patients with rare cancer received expert medical care in the framework of the French National Networks for Rare Cancers in Adults. Among these, 99 cases involved gestational, or pregnancy-associated cancers (PAC) [1]. PAC are rare cancers defined as the occurrence of cancer during pregnancy or within 12 months after delivery. PAC management requires epidemiological evaluation of the pathways of care of these patients, especially as regards the specificities of diagnosis and therapeutic management. The physiological changes during pregnancy can also make diagnosis difficult. Furthermore, treatment of PAC is challenging, with a complex balance required between maternal well-being and the health of the unborn child.

In Martinique (French West-Indies), cancer is the second cause of mortality among females [2]. Developing management of rare cancers in France is a major focus of the 2014–2019 National Cancer Plan. The International Network on Cancer, Infertility and Pregnancy (INCIP) also participates in cancer research in pregnancy to improve the state of knowledge among health professionals and patients.

Few data are available regarding PAC in the Caribbean. This study provides epidemiological data that give a clear description of maternal and neonatal outcomes during the 15-year study period.

## Methods

We performed a retrospective study including all women diagnosed with PAC [cancer diagnosed during pregnancy or within 1 year after delivery] between 1st January 2000 and 31st December 2014 in the University Hospital of Martinique.

PAC cases were identified from Departments of Medical Information according to the International Classification of Disease codes for cancer cross-referenced with codes for pregnancy [3]. Data quality controls performed by the Martinique Cancer Registry enabled validation of all cancer cases. All socio-demographic and clinical data were obtained by consulting medical records in hospital archives.

For each case, socio-demographic (age, marital status, professional activity, familial risk of cancer and comorbidities) and clinical variables (gravidity, parity, term at diagnosis, mode of cancer detection, symptoms, histology of tumor, grade of differentiation, treatment characteristics, pregnancy outcome, neonatal outcome and vital status of the mother) were recorded. Vital status cut-off date was May 21st, 2015. The international TNM Classification was used for cancer staging at diagnosis. This observational study was also based on data from the Martinique Cancer Registry for exhaustiveness of cancer cases. As regards the procedures for cancer registries, all patients are informed by medical doctors and the cancer Registry that cancer cases are recorded in the database of the Registry. This study was also registered with the French national commission for the protection of privacy and personal data (Commission Nationale Informatique et Libertés, CNIL). The Martinique Cancer Registry also participates in the national programme for epidemiological surveillance of cancer in France, under the auspices of the French network of general and specialised cancer registries (FRANCIM). Cancer cases were recorded in strict conformity with the international standards laid down by the International Agency for Research on Cancer (IARC), the French FRANCIM network, and the European Network of Cancer Registries (ENCR). Vital status of patients was obtained from the birth, deaths and marriages registry of each patient’s place of birth up to June 17, 2015. Patient characteristics were described as median or means with standard deviations (SD) for quantitative variables, and as number (percentage) for qualitative variables. Incidence rate was calculated, and the population of pregnant women in the study region during the study period was used as the denominator, i.e. between 2000 and 2014, approximately 112,527 pregnancies in Martinique (total of deliveries and abortions). All analyses were performed using SAS software version 9.2 (SAS Institute Inc., Cary, NC, USA).

## Results

A total of 18 cases were included in this study. The most frequent tumor sites were gynecologic cancers (12 cases, 66.7%): 6 women were diagnosed with breast cancer (33.3%), 3 with ovarian cancer (16.7%) and 3 with cervical cancer (16.7%). Other cases were lymphoma (*n* = 1), brain cancer (*n* = 1), liver cancer (*n* = 1), colorectal cancer (*n* = 1), and skin cancer of the face (*n* = 1). In one case, the primary site was unknown. In our study, the estimated raw incidence of PAC was 0.16/1000 pregnancies during the period 2000–2014. By site, incidence is 0.05/1000 pregnancies for breast cancer and 0.03/ 1000 pregnancies for respectively cervical and ovarian cancer.

Mean age at the time of diagnosis was 35.7 ± 5.4 years, median age was 37.0 years [range 26–44 years]. Table 1 shows the socio-demographic profile of patients with PAC. In 17 cases (94.4%), the diagnosis was made during pregnancy. Only one woman was diagnosed during the post-partum period. Table 2 presents the diagnosis and therapeutic management data.

**Table 1:** Sociodemographic profile of 18 patients diagnosed with pregnancy-associated cancers between 1 January 2000 and 31 December 2014 in Martinique

Variables	N	%
Age (years)		
< 25	0	–
[25–29]	3	16.67
[30–34]	2	11.11
[35–39]	9	50.00
> 40	4	22.22
Gravidity*		
1	3	16.67
2–3	10	55.55
>3	5	27.78
Parity		
0	3	16.67
1–2	12	66.66
>2	3	16.67
Marital status		
Single	4	22.22
Married	10	55.56
Other	4	22.22
BMI**		
Underweight [10–19]	5	31.25
Normal [20–24]	3	18.75
Overweight [25–29]	3	18.75
Obesity [29–80]	5	31.25
Unknown	2	
Profession		
Professionally active	11	73.33
No professional activity	4	26.67
Unknown	3	
Familial risk of cancer		
Yes	4	22.22
Unknown	14	77.78

*Gravidity includes the current pregnancy

**BMI Body Mass Index

**Table 2:** Tumor characteristics, clinical profile, characteristics of diagnosis and therapeutic management

Case number	Tumor site	Histology	Age, y	Gravidity	Parity	Comorbidity	Term at diagnosis	Mode of detection	Type of symptom	TNM	Diagnosis basis	Therapeutic approach
1	Breast cancer	Infiltrating duct adenocarcinoma	32	3	2	High blood pressure	T1	Symptoms	Breast tumefaction	T4N2M1	HP	–
2	Breast cancer	Carcinoma	35	2	1	–	T1	Symptoms	Adenopathy	TXN1cM0	HM	CT^a^
3	Breast cancer	Infiltrating duct carcinoma	39	5	4	–	T3	Symptoms	Motor deficit	T4N3M1	HP	RT/CT/HT
4	Breast cancer	Infiltrating duct carcinoma	40	6	4	–	T2	Symptoms	Breast tumefaction	T1cN0M0	HP	SG/CT
5	Breast cancer	Infiltrating duct adenocarcinoma	43	3	2	–	T3	Symptoms	Breast tumefaction	T4N1M0	HP	CT
6	Breast cancer	Infiltrating duct carcinoma	44	2	1	–	T3	Symptoms	Breast tumefaction	T2N0M0	HP	SG
7	Cervical cancer	Squamous intraepithelial neoplasia	28	1	0	Gestational diabetes	T2	Screening	–	T1N0M0	HP	SG^a^
8	Cervical cancer	Squamous intraepithelial neoplasia	38	3	2	–	T1	Screening	–	T1N0M0	HP	SG
9	Cervical cancer	Invasive adenocarcinoma	39	6	4	High blood pressure	T1	Symptoms	Endocol polyp	T2N0M0	HP	SG^a^/CT-RT/BT
10	Ovarian cancer	Immature malignant teratoma	26	2	1	Cardiac Abnormality	T3	Symptoms	Ovarian mass	T2aN0M0	HP	SG^a^/CT
11	Ovarian cancer	Dysgerminoma	26	3	1	–	T1	Fortuitous	–	T1cN0M0	HP	SG/CT
12	Ovarian cancer	Undifferentiated sarcoma	37	4	2	–	Day 2 PP	Symptoms	Severe pain in pelvix	T3cNXM1	HP	SG/CT
13	HTLV Lymphoma	T-cell large granular lymphocytic leukemia	30	1	0	Polytrauma Chronic	T3	Fortuitous	–	–	Cytology	–
14	Liver cancer	Cholangiocarcinoma	41	5	1	–	T2	Fortuitous	–	T4N1M1	Clinic and paraclinic	–
15	Face skin cancer	Naso-sinusal malignant melanoma	36	3	1	–	T3	Symptoms	Blindness	T4N0M1	HP	CT
16	Colorectal cancer	Neoplasm malignant	35	3	1	–	T2	Symptoms	Anemia	TXNXM1	HM	–
17	Brain cancer	Meningothelial meningioma	37	1	0	–	T1	Symptoms	Adenopathy	M0	HP	–
18	Unknown primitive site	Adenocarcinoma	37	3	1	High blood pressure	T3	Symptoms	Motor paraplegia	TXNXM1	HM	SG/ RT/HT

*BT* brachytherapy, *CT* chemotherapy, *HM* histology on metastasis, *HP* histology on primitive, *HT* hormone therapy, *RT* radiotherapy, *SG* surgery

^a^Treatment during pregnancy

In most cases, PAC was detected in symptomatic individuals (72.2%). In three cases, the detection of cancer was fortuitous and in three other cases, cancer was detected after screening tests. In this study, only one case was multifocal, while seven women had metastases in various sites. Nine women (50.0%) had nodal involvement or initial metastases at the time of diagnosis.

As regards management of PAC, four pregnant women (*cases 2–7–9-10*) received oncological treatment (three were treated by surgery and one by chemotherapy). Among these, only two carried their pregnancy to live birth (*cases 7 and 10*). Eleven women started treatment during the post-partum period and three women died before treatment could be initiated.

In all, 11 pregnancies resulted in a live birth. One woman presented a twin pregnancy associated with cancer. Maternal and neonatal outcomes are presented in Table 3. All cases of caesarean sections before labor, medical termination of pregnancy and voluntary induced abortion (9/18) were motivated by the diagnosis of cancer. Overall, 12 live births from a total of 11 pregnancies (61.1% of PAC) were recorded, with satisfactory APGAR (3 unknown). Seven fetal losses were identified (38.9% of PAC). Mean weight at live birth was 2.7 kg ± 637 g. The rate of preterm birth was 58.3% (7/12 deliveries). Prematurity was spontaneous for two cases and five cases were induced for “therapeutic reasons related to cancer”. The main neonatal outcome observed was prematurity, with six cases of late preterm (34 – 36 weeks and 6 days) and one case of very preterm birth (28 weeks). No other pediatric pathology was described. No child was born after chemotherapy in utero. Six maternal deaths occurred during the study period. The time between diagnosis and death ranged from 9 days to 2 years.

**Table 3:** Pregnancy, maternal and neonatal outcomes

Case	Tumor site	Pregnancy outcome			Neonatal outcome		Maternal follow-up	
		Term at delivery, w	Beginning of labor	Outcome of pregnancy	Fetal weight	APGAR1/5 min	Vital status	Time diagnosis-death,d
1	Breast cancer	14	Induction	MTP	–	–	Dead	9
2	Breast cancer	18	Induction	MTP	250	–	Unknown	–
3	Breast cancer	34	CSBL	Live birth	Unknown	Unknown	Dead	677
4	Breast cancer	20	Induction	MTP	335	–	Alive	–
5	Breast cancer	35	CSBL	Live birth	2680	10/10	Alive	–
6	Breast cancer	38	Induction	Live birth	2490	10/10	Alive	–
7	Cervical cancer	36	Spontaneous	Live birth	3150	10/10	Alive	–
8	Cervical cancer	6	Induction	VIA	–	–	Alive	–
9	Cervical cancer	12	Spontaneous	SM	–	–	Alive	–
10	Ovarian cancer	36	CSBL	Live birth	2540	10/10	Alive	–
11	Ovarian cancer	6	Spontaneous	SM	–	–	Alive	–
12	Ovarian cancer	36	Spontaneous	Live birth	2775	10/10	Dead	24
13	HTLV Lymphoma	39	Induction	Live birth	3795	10/10	Unknown	–
14	Liver cancer	21	Spontaneous	SM	260	–	Dead	30
15	Face skin cancer	35	CSBL	Live birth	2650	6/10	Dead	331
16	Colorectal cancer	28	CSBL	Live birth	1390	8/9	Dead	53
17	Brain cancer	41	Induction	Live birth	2925	10/10	Alive	–
18	Unknown primitive site	Unknown	Unknown	Live birth*2	Unknown*2	Unknown*2	Unknown	–

*CSBL* caesarean section before labor, *d*, days, *min*, minutes, *MTP* medical termination of pregnancy, *SM* spontaneous miscarriage, VIA voluntary induced abortion, *w* weeks

All cases in our study were discussed in cancer staff meetings, and management was discussed with a multidisciplinary team comprising oncologists and gynecologist-obstetricians. Three women received support from a social worker, and 8 women had a consultation with psychologist. Five patients requested expert advice or a second opinion from reference centers in mainland France. The rate of medical transfers organized at the request of professionals was 3/18 (16.6%).

## Discussion

Our study is an overview of PAC management in Martinique.

With 12.3 births per 1000 inhabitants in Martinique, our birth rate is close to the national rate (12.8 per 1000 inhabitants) in 2010. Almost 25% children born in Martinique in 2010 had a mother aged 35 years or more (21.5% of births at a national level). Ten years ago, the respective rates were 19% in Martinique and 16.5% in France [4]. The increasing incidence of PAC is therefore likely due to the older age at which women become pregnant [5]. In the study period [2000–2014], we found 18 women between 26–44 years diagnosed with PAC; with a median age of 37.

In our study, pregnancy-associated-cancer affects approximately 1 in 6250 pregnancies. It is estimated in the literature that cancer complicates between 1/1000 pregnancies and 1/6000 pregnancies [6–8], with the most common malignancies diagnosed during pregnancy being gynecological cancer [9, 10]. Breast cancer is known to be one of the most common cancers occurring during pregnancy (3000–10,000 pregnancies annually) [11]. In Martinique, this is the first cancer site in terms of incidence and mortality [2], and the most common site of PAC in our study.

Ovarian masses complicate pregnancy with an overall incidence of 2.4–5.7% [9]. Grimm et al. [9] even reported a higher incidence, probably explained by more frequent detection thanks to the wider use of ultrasound as a routine antenatal evaluation technique. The asymptomatic character of ovarian cancer makes early diagnosis difficult [12]. Ovarian cancers and cervical cancers were joint second most common in terms of frequency in our study (*n* = 3 for each).

Cervical cancers in women are more frequent in the Caribbean, especially in young women [2]. A recent study conducted in Martinique to describe the epidemiology and survival of cervical cancer based on data from the Martinique cancer registry could be informative for PAC management.

A survey of clinical practices performed by Han et al. showed important differences between physicians in the approach to PAC management [13]. These authors reported that non-academic hospitals tended to prefer termination of pregnancy and refraining from treatment during pregnancy, while, in parallel, academic hospitals had an increased tendency to consider treatment during pregnancy [13]. Interestingly, our results come from a regional university hospital, but correspond to what Han et al. reported to be the preferred strategy for “non-academic centers”, in that we also observed that chemotherapy was not performed in pregnant women and terminations of pregnancy were the preferred option. As yet, there not appear to be a clear move towards a policy of considering the possibility of oncological treatments during pregnancy. According to Kirby [14], “the biggest challenge is educating the public that chemotherapy is possible in pregnancy without harm for the baby.” The literature contains various reports of women who received chemotherapy during pregnancy and who went on to give birth to healthy babies without congenital malformations [12, 15, 16]. In our study, chemotherapy was not the first choice treatment modality for PAC. Only two cases of pregnancies that carried to term underwent surgery during gestation.

In our study, 4 cases (22.2%) of abortion (voluntary or medical) were mentioned with maternal cancer as the motive. For invasive cancer, Van Calsteren and al [10] described the outcomes of 215 cases of PAC and reported 30 cases (14.0%) of pregnancy terminations, with the main indication (29/30) being the maternal malignancy. Several studies have shown that interruption of pregnancy does not improve maternal prognosis but is indicated when cytotoxic treatment is an emergency in the first trimester [14, 17, 18]. National and international guidelines have been published for the management of several sites of PAC [5] [19, 20]. However, in the absence of large randomized trials, it is difficult to know how best to manage these women. Each case needs to be addressed individually, and should be discussed case-by-case, taking into account the term of the pregnancy, the patient’s wishes and the tumor stage. In Martinique, multidisciplinary cancer staff meetings bring together oncologists and obstetric gynecologists. Neonatologists, onco-psychiatrists and social workers can be invited to participate in these meetings with a view to better management of PAC. Ferrere and Wendland indicated that PAC is a situation that incurs a major risk of anxiety and depressive symptoms, and there is much to be gained from evaluation of psychopathological risks in this context [21]. In our study, eight women had psychological consultations.

In most studies and reviews [22–24], PAC is considered to have an unfavorable prognosis due to delayed diagnosis and limitations in oncologic therapy. In our study, 9 women had nodal involvement or initial metastases at diagnosis. Any delay in treatment may unacceptably worsen the maternal prognosis. The major neonatal outcome in our series was preterm birth, with 58.3% of viable deliveries, and this rate is similar to another study [10]. Indeed, Van Calsteren et al. [10] concluded that prevention of iatrogenic prematurity appears to be an important part of the treatment strategy. The real incidence of PAC in Martinique is difficult to assess because of the lack of a specific PAC registry for exhaustive case collection. The existence in our study of only a single reported case of PAC during the postpartum period could suggest under-reporting of incident cases. The number of cases in our study is dependent on the quality of the coding of medical records. According to the study conducted by Han et al. [13], 75% of European physicians interviewed were interested in registering patients anonymously in a centralized database. A Caribbean Observatory for Rare Cancers could collect and follow a larger number of PAC and other rare cancers. This observatory could constitute an inter-regional center of expertise for the French National Network for health professionals.

The French national PAC network (“Cancers Associés à la Grossesse”) created in 2008 in Metropolitan France, under the auspices of the National College of Obstetricians and Gynecologists, is a platform for coordination and cooperation between clinical hospital units. It was created to optimize and homogenize care of patients throughout France. With an estimated incidence of 500 new cases per year in 2013, only 99 new cases had been registered, indicating a low coverage rate of 19.8% [25]. The Caribbean Observatory for Rare Cancers could help in increasing this coverage rate.

### Strengths and weaknesses of the study

To the best of our knowledge, this is the first study to focus on maternal and neonatal outcomes after PAC in the French West-Indies (Martinique). In the Caribbean archipelago, few studies have been conducted on this topic to date [26, 27]. Our results can be considered as a representative sample of the target population in Martinique, since management of PAC is mainly organized in University Hospital of Martinique, and all cases of PAC were addressed to our university hospital referral centre for care. Cross-referencing of data from the Department of Medical Information with the data from the Cancer Registry, as well as examination of source medical files enabled us to exclude any cases not corresponding to cancers in pregnancy.

The main limitation of this cross-sectional study is the methodological difficulty in identifying patients for case collection, since case identification depends directly on the accuracy of the data extracted from medical informatics by the Department of Medical Information. In addition, there may be some selection bias due to patients living in Martinique who choose to travel elsewhere (in particular, to mainland France) for treatment, either for personal reasons or because they are encouraged to do so by their physician. However, these limits would likely lead to under-representation of the true number of cases, and therefore, underestimation of incidence.

The number of cases in our study also remains directly dependent on the quality of the coding of records by medical professionals. The high rate of transfers increases the proportion of missing data. The detection of a single case of PAC during the postpartum could suggest under-reporting of incident cases.

## Conclusion

Collaboration with this national network could be helpful for the organization of patient transfers to metropolitan France and post-transfer monitoring. Cancer management during pregnancy is a challenge for the French West Indies territories. Close cooperation with the International Network on Cancer, Infertility and Pregnancy could help to promote PAC research.

## Abbreviations

CNIL: Commission Nationale Informatique et Libertés
ENCR: European Network of Cancer Registries
FRANCIM: France Cancer Incidence et Mortalité
IARC: International Agency for Research on Cancer
INCIP: International Network on Cancer, Infertility and Pregnancy
PAC: Pregnancy-associated-cancer
